# Effects of Mindfulness-Based Cognitive Therapy on Peripheral Markers of Stress and Inflammation in Older-Adults With Depression and Anxiety: A Parallel Analysis of a Randomized Controlled Trial

**DOI:** 10.3389/fpsyt.2021.804269

**Published:** 2021-12-24

**Authors:** Claudia Belliveau, Corina Nagy, Sophia Escobar, Naguib Mechawar, Gustavo Turecki, Soham Rej, Susana G. Torres-Platas

**Affiliations:** ^1^McGill Group for Suicide Studies (MGSS), Douglas Mental Health University Institute, Montreal, QC, Canada; ^2^Geri-PARTy Research Group, Jewish General Hospital, Montreal, QC, Canada; ^3^Department of Psychiatry, McGill University, Montreal, QC, Canada

**Keywords:** depression, anxiety, geriatric, mindfulness based cognitive therapy, inflammation, stress, biomarkers

## Abstract

**Background:** Depression and anxiety are prevalent in older-adults and often difficult to treat: up to 55% of patients are unresponsive to pharmacotherapy. Mindfulness-Based Cognitive Therapy (MBCT) is a promising treatment, however, its biological mechanisms remain unknown in older-adults.

**Methods:** We examined if, in older-adults, decreased depression and anxiety symptoms after MBCT are associated with changes in the expression levels of C-reactive protein, Interleukin-1β, Monocyte chemoattractant protein-1 and mineralocorticoid receptor compared to treatment as usual (TAU). Older-adults (age ≥60) with depression and anxiety were randomized to MBCT or treatment as usual. Gene expression levels from blood samples were measured using quantitative polymerase chain reaction (*n* = 37) at baseline and after 8-weeks of MBCT or TAU.

**Results:** As previously published, we found a significant reduction in symptoms of depression F (1, 35) = 10.68, *p* = 0.002, partial η^2^ = 0.23 and anxiety F (1, 35) = 9.36, *p* = 0.004, partial η^2^ = 0.21 in geriatric participants following MBCT compared to TAU. However, the expression levels of measured genes were not significantly different between groups and were not associated with changes in depression and anxiety symptoms.

**Conclusion:** Our results suggest that the symptom reduction following MBCT in older-adults may not be accompanied by changes in the stress-response and inflammatory pathways. Future research should address other potential biological alterations associated to MBCT that may be responsible for the reduction of symptoms.

## Background

Depression is the leading cause of disability worldwide and is often comorbid with anxiety symptoms (1). Approximately, 10% of geriatric patients in primary care suffer from depressive disorders (2, 3), and almost 50% of those suffer from co-occurring symptoms of anxiety (3, 4). An estimated 50–55% of patients with depression in late-life will be resistant to pharmacotherapies (5, 6) and one-on-one psychotherapy may not be an option due to a limitation in financial resources. Over the past few decades Mindfulness-Based Interventions (MI) have become more common place in the treatment of mental health disorders (7–11). These interventions are often used as adjuncts to medication or as a replacement for traditional psychotherapy (8). MI provide patients with mastery of objective observation, awareness, and acceptance among other stress management techniques (12). Mindfulness-Based Cognitive Therapy (MBCT) is a form of MI that combines mindfulness with cognitive behavioral techniques to reduce emotional reactivity (13) offered in a group setting by a therapist (14, 15). Evidence suggests that MBCT, is effective at reducing the symptoms of depression and anxiety in primary care (7, 14–19) and preventing relapse in populations that are more at risk for depression (16). In geriatric populations, the evidence is scarce, yet, in the recent randomized controlled trial (RCT) performed by our group, we found a significant reduction in symptoms of depression and anxiety and an increased quality of life after 8-weeks of MBCT when compared to treatment as usual (TAU) (20).

Depression and anxiety have consistently been linked with increases of inflammation and stress markers (21, 22), particularly in senescence (23). Interestingly, in different inflammatory conditions such as fibromyalgia (24) and chronic pain (25), MI have shown to be particularly effective in decreasing levels of low-grade inflammation markers. Studies in younger adults attempting to elucidate the physiological mechanisms behind symptom reduction in depression and anxiety after MI (26) have targeted the stress and inflammatory systems (27, 28) given their differential regulation in response to pharmacological treatment outcomes (16, 29, 30) and their constant implication in the etiology of depression and anxiety (31, 32). However, the results are heterogeneous and inconclusive in younger adults (33–35), and virtually inexistent in older-adults suffering from depression and anxiety (36). For instance, in peripheral blood samples of younger adults suffering from major depressive and anxiety disorders undergoing MI, there were no significant differences in the protein levels of C-reactive protein (CRP) (37), interleukin-8 (IL)-8 and high-sensitivity C-reactive protein (hsCRP) (34) however, the levels of endothelial growth factor (EGF) (34), adrenocorticotropic hormone (ACTH), tumor necrosis factor alpha (TNF-α) and IL-6 (38) levels were significantly decreased from baseline to post-intervention when compared to controls. Similarly, in salivary samples significant decrease in IL-6 and TNF-α were reported in depressed participants but the levels of cortisol were similar in patients remitted from major depressive disorder when compared to controls (39).

In older-adults, evidence suggests that MI downregulated the NF-κB-associated gene expression profile in circulating leukocytes at post-treatment in lonely adults compared to the control group (36). The levels of hs-CRP in older-adults with mild cognitive impairment were significantly decreased after 9-months of MI when compared to the active control group (40). Taken together, this evidence suggests that MI could mediate the levels of some stress and inflammatory markers in older-adults. To our knowledge, there are no studies that have assessed the association of MI with stress and inflammatory gene expression in older-adults suffering from depression and anxiety in primary care. Therefore, in the present study, we aim to first examine, compared to TAU, whether MBCT is associated with changes in the expression levels of genes involved in the stress and inflammation response namely: CRP, IL-1β, Monocyte chemoattractant protein-1 (MCP1) and mineralocorticoid receptor (NR3C2). Then, investigate if these changes are associated with reductions in depression and anxiety scores. The present study was designed under the umbrella of our previously published RCT, where we collected blood samples from older-adults that underwent 8-weeks of MBCT and showed significant decrease in symptoms of depression and anxiety when compared to controls undergoing TAU (20).

## Methods

### Study Design, Participants, and Setting

This is a pre-planned parallel analysis of a RCT investigating the effects of MBCT on depression and anxiety symptoms in older-adults (20). Patients aged 60 years and above, with depression and/or anxiety symptoms were recruited from five primary care centers in Montreal, Canada, between September 1st, 2016 and December 20th, 2017. Participants were included in the study if they had a score of ≥10 (moderate depression/anxiety) on the Patient Health Questionnaire (PHQ-9) and/or General Anxiety Disorder-7 (GAD-7). Participant were excluded if they presented acute psychotic symptoms, severe personality disorder/ unable to function in a group setting, acute suicidal ideations or intent (20). Patients who fulfilled selection criteria and provided written consent were asked to fill out self-report questionnaires and provide blood samples collected by qualified nurses. Participants were randomized 1:1 into two groups: MBCT or TAU. Randomization and coding were performed by a third party not involved in assessment and recruitment using a web application (randomizer.org). Biological experiments were conducted by an investigator blind to the participants' group allocation, who did not assist in MBCT intervention and was not involved in randomization. The extensive methods are published elsewhere (20).

### MBCT Intervention

The MBCT intervention involved weekly 2 h group sessions for 8 weeks. The therapeutic intervention followed Segal et al. manualized protocol (15) with some adaptations made to accommodate the needs of the older-adult participants. Briefly, participants learned mindfulness techniques, followed by group discussions around how to apply awareness, non-judgment, and acceptance to daily life. Between sessions, participants practiced daily for at least 15 min. Adaptations included: (1) reducing time of body scan meditation, (2) replacing yoga mats with chairs during meditation practices, (3) performing seated yoga using modified movements, (4) encouraging participants to modify postures and pillows to promote well-being and safety, (5) slowing down the pace of walking meditation, (6) ensuring all participants can hear and understand by projecting the voice and (7) taking more frequent breaks. More detailed explanations are published elsewhere (20).

### TAU Control

Participants in TAU received routine care or treatment as usual in the primary care center, which includes antidepressant medication and/or support with a primary care team member (e.g., social worker, nurse, and/or psychologist).

### Collection of Biological Samples

Following informed consent from the participants, regardless of group, samples were collected by qualified nurses at the main primary care study site (CLSC Benny Farm). Blood samples were transported to the Douglas Mental Health University Institute, Montreal, Canada, on ice, up-to 2 h after collection and processed following a previously published protocol for the cryopreservation of peripheral blood mononuclear cells (41). Briefly, blood samples were diluted (1:1 PBS) and run through a Ficoll-Paque PLUS (GE Healthcare, Sweden) gradient centrifuged at 400 g for 35 min at room temperature. The plasma layer was discarded and the lymphocyte/monocyte layer was collected and washed with PBS to remove platelets. Samples were resuspended in RPMI-1640 Medium (Sigma-Aldrich, USA) with 10% dimethyl sulfoxide (DMSO) (Sigma-Aldrich, USA) as the anti-freeze agent and stored in an −80°C freezer until use.

### RNA Extraction

Samples were thawed and washed with PBS to remove RPMI-1640 Medium and 10% DMSO. Then total RNA was extracted using the Direct-zol RNA MiniPrep kit (Zymo, USA) including DNase treatment following manufacturer's guidelines. RNA quality was measured using Agilent 2200 TapeStation which assigns an RNA integrity score (RINe) to each sample based on RNA degradation; RINe is scored out of 10, with 10 being the best quality. RNA integrity did not vary significantly between groups (TAU = 7.0 ± 1.1; MBCT = 7.2 ± 1.2; *p* > 0.05). A NanoDrop Spectrophotometer was used to quantify the concentration of RNA in each sample, the average concentration did not vary significantly between groups (TAU = 110.3 ± 47.4 ng/μl; MBCT = 103.0 ± 58.3 ng/μl; *p* > 0.05). Each sample had a 260/280 nm ratio of ~2 indicative of pure RNA.

### Absolute Quantification of Gene Expression

Next, cDNA (normalized to 20 ng/μl) was synthesized using iScript Reverse Transcription Supermix for RT-qPCR (Bio-Rad, USA) according to manufacturer's protocol. Primer pairs against genes of interest were designed using NCBI Primer-BLAST and optimized for PowerUp SYBR Green (ThermoFisher, USA) quantitative polymerase chain reaction (qPCR) [CRP: CAGACAGACATGTCGAGGAAGG; TCCGTGTAGAAGTGGAGGCA (125 bp). IL1-β:GCTGGAGAGTGTAGATCCCAAA; CTGCTTGAGAGGTGCTGATGT (140 bp). MCP1: CTGCTTGAGAGGTGCTGATGT; CTTGAAGATCACAGCTTCTTTGG (113 bp). NR3C2: GCAAAGGCAATACCAGGTTTCA; CAGGAGCAAAACACAGCAGG (145 bp).ACTIN-β: AGACCTGTACGCCAACACAG; GCGCTCAGGAGGAGCAATG (133 bp)]. Endogenous controls (RAG-1, ACTIN-β, GAPDH and ATP-ase primers) were tested for least variability between samples, and ACTIN-β was chosen and run with each sample for each target. All targets were run in triplicate, and all plates were run within 24 h of each other on the Applied Biosystems QuantStudio 6 Flex Real-Time PCR instrument. Triplicates were analyzed and samples with C_T_ SD > 0.03 were omitted. Quantity mean of target was normalized to ACTIN-β quantity for that sample.

### Gene Targets

The genes of interest were chosen based on literature suggesting depression and anxiety in the older-adult population are preceded by increased inflammation. The primary target of interest was CRP, a commonly investigated circulating marker of inflammation and tissue damage with increased levels being associated with depression (42) and anxiety in older-adults (31). Secondary targets of interest included genes that are commonly investigated in depression and anxiety literature however are less consistently linked. MCP1 a chemokine that regulates other cytokines, has been found to be modified by antidepressants (43) and secreted to recruit monocytes in the central nervous system. IL-1β, which implication in depression and anxiety has been a subject of debate due to heterogenous samples, (44), is found to be elevated in depressed older-adults (>60 years) (45). Finally, NR3C2 was examined as the hypothalamic-pituitary-adrenal (HPA) axis dysregulation and inflammation are tightly intertwined in depression (46).

### Depression and Anxiety Outcome Measures

Depression and anxiety were measured using the PHQ-9 and GAD-7. Both are validated measures with high internal reliability (measured using Crohnbach's α > 0.8) (47–49).

### Data Analysis

Baseline demographics and clinical information were characterized using descriptive statistics [% (n), mean (± standard deviation)]. Normality was assessed using the Shapiro-Wilk test and then compared between groups using two-sided independent *t*- and chi squared tests, as appropriate. Changes in PHQ-9 (depression) and GAD-7 (anxiety) scores were assessed using repeated measures analysis of variance comparing the group undergoing MBCT to TAU. Grubb's test was used to detect outliers in target gene expression analyses, only one significant outlier per group was removed for each target. For the 1^st^ research question, we examined whether expression levels of our targeted genes changed after treatment using a mixed model for repeated measures using the Restricted Maximum Likelihood (REML) method on GraphPad Prism 9.0.0. For the 2^nd^ research question, we used correlation to assess whether the changes in PHQ-9 and GAD-7 scores were associated with variation in target gene expression. Both the Statistical Package for Social Sciences (SPSS) version 27 (IBM, Chicago, IL) and GraphPad Prism 9.0.0 (GraphPad Software, San Diego, California USA) were used to perform data analyses. Effect size was calculated using an online calculator offered by Psychometrica (50). A two-tailed *p* < 0.05 was considered statistically significant.

## Results

**Figure 1:**
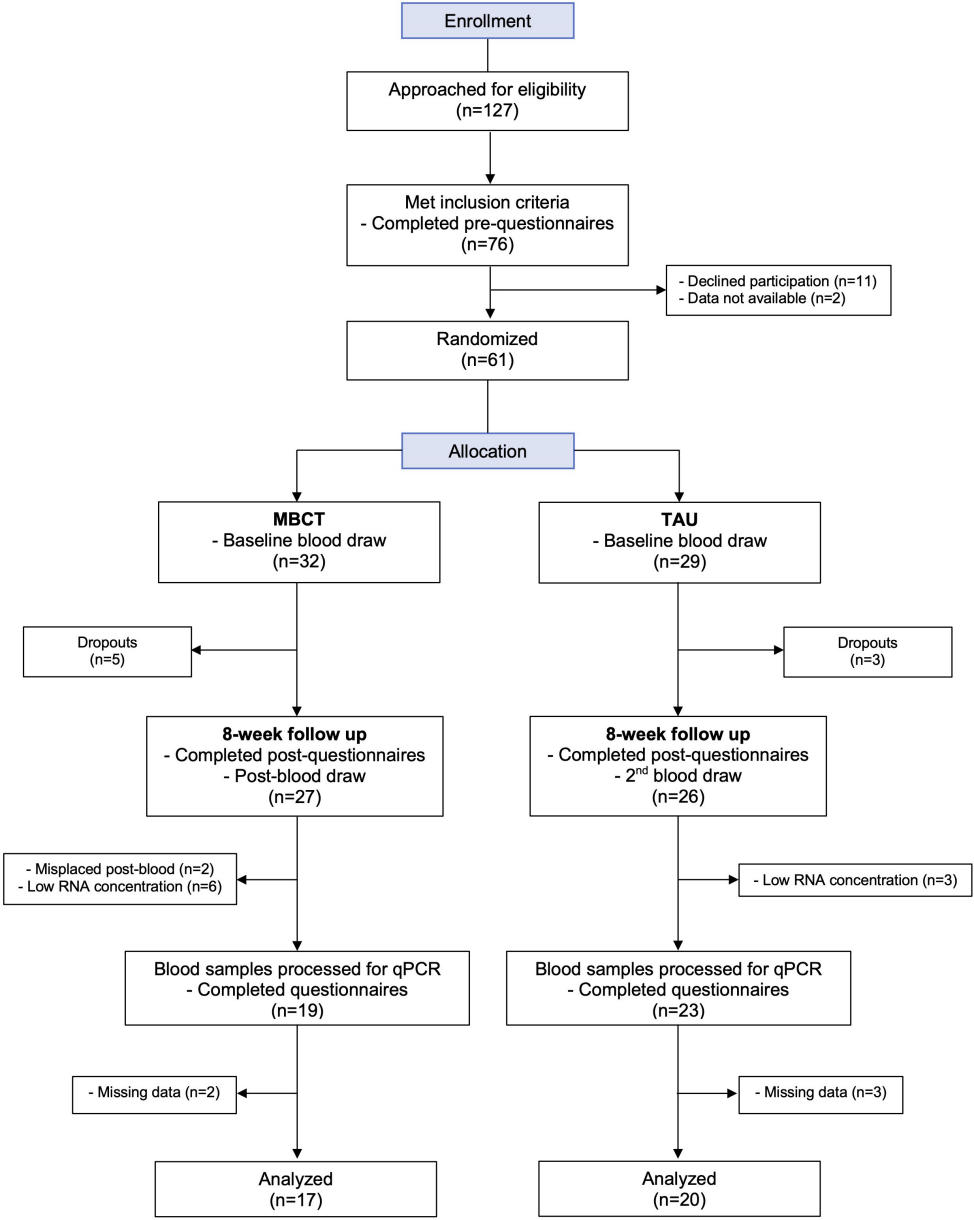
Participant flow chart. MBCT, Mindfulness Based Cognitive Therapy treatment group; TAU, Treatment as usual group.

In our previous study (20), a total of 76 primary care individuals were eligible for participation, and 61 patients were successfully enrolled. These 61 participants answered pre-questionnaires and, for this parallel study, 100% consented to baseline blood draws taken by nurses at CLSC Benny Farm. Participants were randomized and allocated to MBCT (*n* = 32) or TAU (*n* = 29). During the 8-week treatment period there were 8 participants that dropped out for non-specified reasons (Figure 1). A total of 86.9% (53/61) of participants completed post-questionnaires and blood draws at the 8-week follow up (84.4% MBCT = 27/32 and 89.7% TAU = 26/29). A few blood samples were missing or had too low of an RNA concentration for the following steps, leaving 42 participants with complete profiles (MBCT = 19, TAU = 23) that were processed for qPCR. A total of 88.1% of participant samples (37/42) were included in analysis (MBCT = 17, TAU = 20), some samples were excluded from analysis due to missing data (Figure 1). The mean age of participants included in this analysis was 68 ± 6.2 years, with the oldest participant being 85 years old (Table 1). Females comprised 65% of the sub-sample. Participants in MBCT and TAU groups did not differ significantly with respect to baseline characteristics (*p* > 0.05; Table 1).

**Table 1:** Baseline demographics, clinical and target gene expression data.

**Participant data**	**Total sample** **(***n*** = 37)**	**MBCT** **(***n*** = 17)**	**TAU** **(***n*** = 20)**
	**Mean (±SD) %** **(***n***)**	**Mean (±SD) %** **(***n***)**	**Mean (±SD)%** **(***n***)**
**Demographic information**			
Female	65% (24)	76.5% (13)	55% (11)
Age, yr	68 ± 6.2	67.8 ± 6.8	68.1 ± 5.9
**Medical history**			
Number of medical problems	2.3 ± 1.5	2.2 ± 1.1	2.4 ± 1.7
Number of current medications	4.31 ± 3.7	4.53 ± 4.4	4.11 ± 2.9
**Mental health information**			
Anxiety diagnosis	64.9% (24)	52.9% (9)	75% (15)
Depression diagnosis	59.5% (22)	52.9% (9)	65% (13)
Others diagnosis	10.8% (4)	17.7% (3)	5% (1)
Number of years diagnosed	11.5 ± 10.5	11.6 ± 11.5	11.4 ± 10.5
Number of years symptomatic	21.8 ± 20.5	17.7 ± 21.8	25.1 ± 19.5
Number of current psychotropics	1.06 ± 1.0	1.06 ± 1.2	1.05 ± 0.9
Psychotropic Medications	56.8% (21)	47.1% (8)	65% (13)
Antidepressants (ATD)	51.4% (19)	41.2% (7)	60% (12)
Hypnotic/sedatives	16.2% (6)	23.5% (4)	10% (2)
ATD and hypnotic/sedatives	16.2% (6)	23.5% (4)	10% (2)
Antipsychotics	8.1% (3)	5.9% (1)	10% (2)
Other	2.7% (1)	5.9% (1)	0% (*n* = 0)
Current mental health follow-up	45.9% (17)	41.2% (7)	50% (10)
**Questionnaire scores**			
Depression score (PHQ-9)	16.03 ± 5.3	16.00 ± 5.9	16.05 ± 5.0
Anxiety score (GAD-7)	12.27 ± 4.6	12.18 ± 3.8	12.35 ± 5.3
**Target gene expression**			
CRP^*^	1.09 ± 0.96	0.98 ± 0.98	1.16 ± 0.96
IL-1β^*^	0.50 ± 0.28	0.41 ± 0.17	0.56 ± 0.33
MCP1^*^	1.11 ± 1.00	1.11 ± 1.23	1.10 ± 0.73
NR3C2^*^	1.20 ± 0.74	1.18 ± 0.97	1.21 ± 0.59

* *Normalized value (mean quantity of target/ mean quantity of ACTIN-B)*.

*No significant differences were found between TAU and MBCT groups in any of the demographic measurements*.

**Figure 2:**
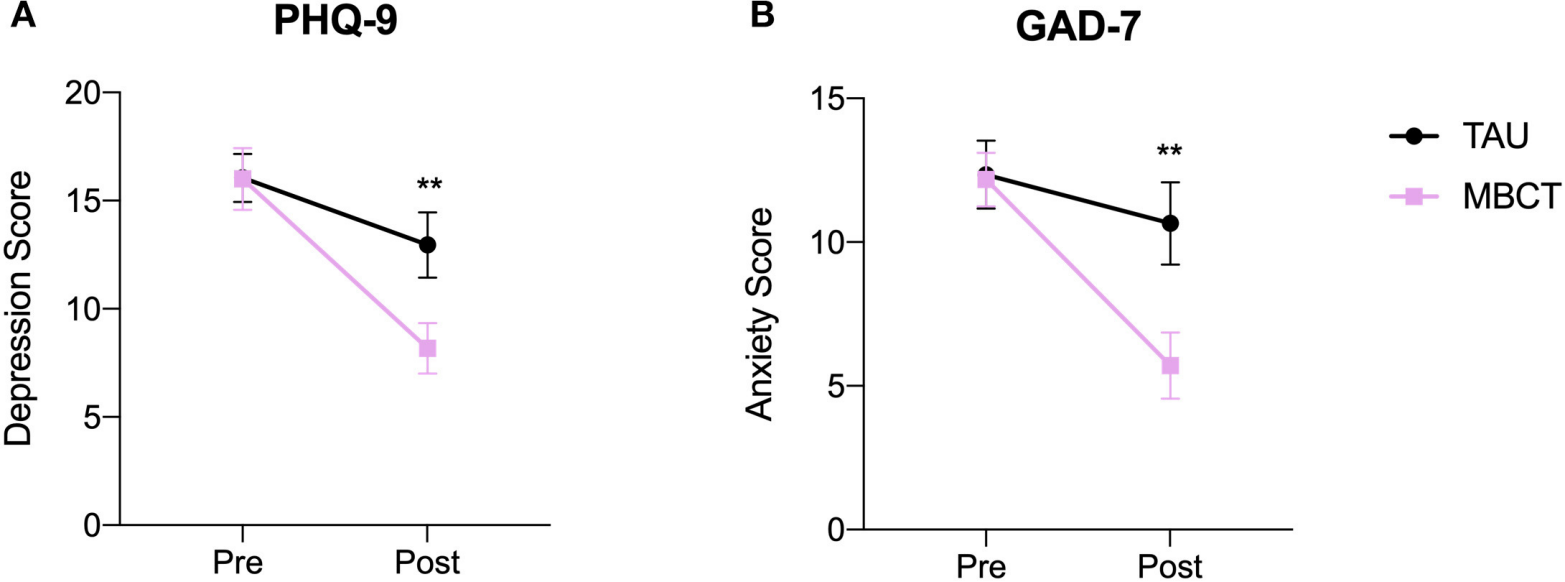
Participants undergoing 8-week MBCT presented a significant reduction in depression **(A)** and anxiety **(B)** scores compared to TAU. ** *p* < 0.01, error bars: standard deviation. MBCT, Mindfulness Based Cognitive Therapy treatment group; TAU, Treatment as usual group; PHQ-9, Patient Health Questionnaire 9 items; GAD-7, General Anxiety Disorder questionnaire 7 items.

First, we assessed whether this sub-sample of participants (*n* = 37) presented a significant decrease in depression and anxiety scores after 8-weeks of MBCT when compared to TAU as similarly reported in our previous RCT (*n* = 61) (20). Indeed, the symptoms of depression and anxiety were significantly decreased after 8-weeks of MBCT treatment when compared to TAU assessed with their changes in PHQ-9 score F *(1, 35)* = *10.68, p* = *0.002, partial η*^2^ = *0.23* (MBCT 16.0 ± 5.9 vs. 8.2 ± 4.8 and TAU 16.1 ± 5.0 vs. 13.0 ± 6.7) and GAD-7 score *F (1, 35)* = *9.36, p* = *0.004, partial η*^2^ = *0.21* (MBCT 12.2 ± 3.8 vs 5.7 ± 4.8 and TAU 12.4 ± 5.3 vs 10.7 ± 6.4) (Figure 2).

**Figure 3:**
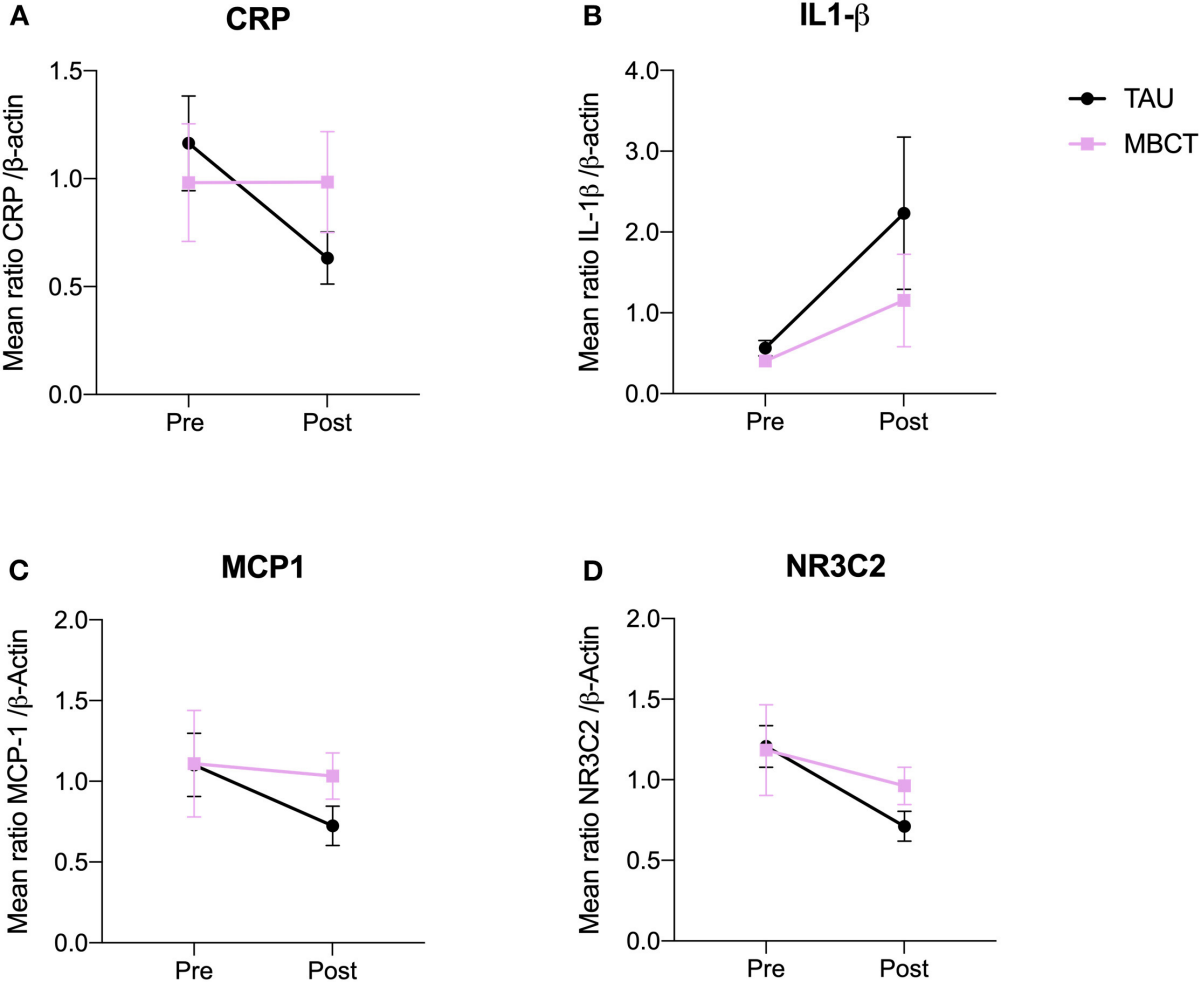
Compared to TAU, MBCT is not accompanied by changes in the levels of CRP **(A)**, IL1-β **(B)**, MCP1 **(C)**, NR3C2 **(D)**. Error bars indicate standard deviation. MBCT, Mindfulness Based Cognitive Therapy treatment group; TAU, Treatment as usual group; CRP, C-reactive protein; IL1-β, Interleukin 1-Beta; MCP1, Monocyte chemoattractant protein-1; NR3C2, mineralocorticoid receptor.

Our first objective was to assess if, compared to TAU, the symptom reduction of the MBCT group was accompanied by reductions in the expression of the stress and immune system activation markers CRP, IL1-β, MCP1, NR3C2, at 8-week follow-up. We found no significant differences between groups in expression levels of CRP *F (1, 54)* = *1.36 p* = *0.25, d* = *0.41* (Figure 3A), IL1-β *F (1, 19)* = *0.54 p* = *0.47, d* = *0.32* (Figure 3B), MCP1 *F (1, 20)* = *0.47 p* = *0.50, d* = *0.25* (Figure 3C), NR3C2 *F (1, 21)* = *0.92 p* = *0.35, d* = *0.34* (Figure 3D).

Our second question assessed the relationship between the change in expression levels of the genes of interest and reduction of depression and anxiety symptoms. No significant correlations were found between change in targets and change in PHQ-9 or GAD-7 scores (*p* > 0.05).

There was no significant correlation between the number of medications, or number of mental health medications with change in PHQ-9/GAD-7 score or change in expression levels in either group (*p* > 0.05).

## Discussion

This is the first study, to our knowledge, that investigates gene expression levels of stress and inflammation markers in the peripheral blood of depressed and anxious older-adults after 8-weeks of MBCT. Despite our confirmation of a significant decrease in depression and anxiety symptoms following MBCT (*n* = 37), which is consistent with our previous findings (20) and others in younger adults (7, 16, 17). Here we found that: (1) the MBCT group did not present significant changes in differential gene expression levels of CRP, IL1-β, MCP1 and NR3C2 and (2) changes in gene expression levels were not correlated with improvements in depression and anxiety symptoms.

Evidence suggests that depression and anxiety are accompanied by a differential regulation of inflammatory cytokines, chemokines and stress hormones such as CRP, cortisol (51) and NR3C2 (52). Younger adults suffering from depression present a significant decrease in the peripheral levels of IL-6, TNF-α, IL-10, and MCP1 after pharmacological treatment with antidepressants. This suggests that symptom reduction acts through the regulation of inflammatory pathways (16, 29, 30). In younger adults, functional genomic studies indicate gene expression changes in leukocytes along with reduction in telomere shortening after MI (53). Therefore, it is natural to hypothesize that the reported MBCT-related symptom reduction in depression and anxiety is also mediated through inflammatory pathways. A few research groups have worked under this hypothesis, and the results of their studies in younger adults found that exposure to MBCT resulted in a significant reduction in EGF but not in the levels of circulating IL-8 and CRP in subjects with depression and anxiety when compared to controls (34). Additionally, there was no significant difference in the levels of salivary cortisol of remitted patients from recurring depression (39) when compared to controls. However, another study showed a significant decrease in salivary levels of IL-6 and TNF-α after 4 weeks of MI when compared to controls (54).

Our results in the context of these investigations in older-adults, showed no significant differences in the CRP levels similar to the results from a RCT by Memon and colleagues in a trial with younger adults, that shows the symptom improvement after 8-week MBCT is not associated with a differential gene expression of CRP (34). We did not find significant differences in IL1-β, MCP1 and NR3C2. However, it is possible that the levels of these and other cytokines/chemokines, such as IL-6 and TNF-α, may be dysregulated in the brain or blood of older-adults with depression and anxiety; since other cognitive therapies such as CBT, have shown to reduce symptoms of depression and the levels of IL-6 and TNF-α (55). It is also possible that the mechanism of action of MBCT differs between younger and older-adults.

### Strengths and Limitations

A major strength of this study is that this clinical intervention was combined with psychological assessment and biological experimentation to better understand the link between the treatment outcomes and mechanisms. Additionally, this is the first study to examine the expression level of genes related to stress and inflammation in a population of depressed and anxious older-adults after 8-weeks of MBCT. Moreover, we have a below average dropout rate 13% (8/61) for a psychotherapy RCT of this size (56) and 100% of enrolled participants consented to blood draws in addition to the battery of questionnaires.

Our study has a few limitations. Although we were still able to detect changes in depression and anxiety scores after 8-weeks of MBCT with our sub-sample (*n* = 37) we were also expecting to find an accompanying downregulation in the expression of the chosen markers, as it has been shown in younger adults and inflammatory and stress markers tend to be increased in depression and anxiety. While our sample size is comparable to other RCTs examining biological data in lonely older-adults (36) after MI, it is below average sample size of studies including younger adults (16). The modest sample size in this study is likely influencing the weak effect size of our results at the gene expression level. Other studies with larger sample sizes need to be conducted to assess the effects of MBCT on various stress and inflammatory markers in older-adults.

Although our original battery of questionnaires included many important measurements including quality of life (EuroQol 5-D), depression and anxiety symptoms (PHQ-9/GAD-7), and quality of sleep (Athens Insomnia Scale); future studies should include measurements that track stress levels throughout the course of treatment (Perceived Stress Scale or Perceived Stress Questionnaire). These questionnaires would allow for an understanding of perceived stress and improve the understanding of how MBCT changes thought patterns and reduces the perception of stress even when stressors have not been removed directly.

Here, gene expression was evaluated using qPCR immediately after the 8-week treatment; we do not rule out the possibility that these genes are differentially regulated on a larger temporal scale or post-translationally. Indeed, in a recent study of mindfulness awareness practice in mild cognitively impaired older-adults, significant decreases in stress biomarker levels were seen at the 9-month mark while changes in inflammatory markers were seen after 3-months of weekly treatment (40). Moreover, our panel of chosen genes did not show differences between groups, however, it is possible that other genes in the stress and inflammatory pathway may be differentially regulated. To address this limitation, the panel of genes should be expanded to include those that have previously been involved in the pathophysiology of depression and anxiety such as IL-6, IL-10, NFκB, and IFNγ. Furthermore, whole genome transcriptomic analyses may elucidate new targets and pathways involved in the biological mechanisms of MBCT. Interestingly, accumulating oxidative stress during the process of aging influences the efficiency of translation and the stability of mRNA (57) so future studies should investigate both mRNA expression and protein levels to ensure the entire picture is captured.

## Conclusion

Overall, the results of our pre-planned parallel analysis showed that, in this population, MBCT did not significantly alter the gene expression of CRP, IL1-β, MCP1, NR3C2. These elements of the stress/inflammation pathway may not be the sole mechanism underlying symptom improvement of depression and anxiety after MBCT in older-adults. Future research needs to address other potential biological alterations associated to MBCT that are responsible for the reduction of symptoms. A complete analysis of the different elements of the stress/inflammatory pathway at the mRNA and protein level need to be assessed. This study should be replicated in a longitudinal clinical trial with an active control group, with more participants and using more advanced methods such as RNA sequencing or proteomics that can survey all the genes/proteins involved in the stress and inflammation pathways.

## Data Availability Statement

The original contributions presented in the study are included in the article/supplementary materials, further inquiries can be directed to the corresponding author.

## Ethics Statement

The studies involving human participants were reviewed and approved by Jewish General Hospital Ethics Committee. The patients/participants provided their written informed consent to participate in this study.

## Author Contributions

CB: data curation, formal analysis, investigation, writing – original draft preparation, and visualization. CN: conceptualization, methodology, supervision, and writing – review and editing. SE: investigation and writing – review and editing. NM: supervision and writing – review and editing. GT: resources and supervision. SR: resources, writing – review and editing, supervision, and funding acquisition. SGTP: conceptualization, methodology, writing – review and editing, supervision, and project administration. All authors contributed to the article and approved the submitted version.

## Funding

SR receives Early Career salary support from the Fonds de Recherche Quebec Santé and owns shares of Aifred Health. The funding was used to obtain lab materials needed for the molecular analysis of the blood samples.

## Conflict of Interest

The authors declare that the research was conducted in the absence of any commercial or financial relationships that could be construed as a potential conflict of interest.

## Publisher's Note

All claims expressed in this article are solely those of the authors and do not necessarily represent those of their affiliated organizations, or those of the publisher, the editors and the reviewers. Any product that may be evaluated in this article, or claim that may be made by its manufacturer, is not guaranteed or endorsed by the publisher.

## Abbreviations

MBCT: Mindfulness-Based Cognitive Therapy
TAU: treatment as usual
RCT: randomized controlled trial
MI: Mindfulness Based Interventions
CRP: c-reactive protein
IL: interleukin
hsCRP: high-sensitivity c-reactive protein
EGF: endothelial growth factor
ACTH: adrenocorticotropic hormone
TNF- α: tumor necrosis factor alpha
MCP1: Monocyte Chemoattractant Protein-1
NR3C2: mineralocorticoid receptor
PHQ-9: Patient Health Questionnaire
GAD-7: General Anxiety Disorder-7
DMSO: dimethyl sulfoxide
RINe: RNA integrity score
qPCR: quantitative polymerase chain reaction
HPA: hypothalamic-pituitary-adrenal
REML: Restricted Maximum Likelihood
SPSS: Statistical Package for Social Sciences.
